# AcumenTM hypotension prediction index guidance for prevention and treatment of hypotension in noncardiac surgery: a prospective, single-arm, multicenter trial

**DOI:** 10.1186/s13741-024-00369-9

**Published:** 2024-03-04

**Authors:** Xiaodong Bao, Sathish S. Kumar, Nirav J. Shah, Donald Penning, Mitchell Weinstein, Gaurav Malhotra, Sydney Rose, David Drover, Matthew W. Pennington, Karen Domino, Lingzhong Meng, Mariam Treggiari, Claudia Clavijo, Gebhard Wagener, Hovig Chitilian, Kamal Maheshwari, The HPI Study Team, Kathryn Cody, Ariel Muller, Anna L. Christensen

**Affiliations:** 1 Anesthesia, Critical Care and Pain Medicine, Massachusetts General Hospital, Boston, MA USA; 2 Department of Anesthesiology, University of Michigan Medical School, Ann Arbor, MI USA; 3 Department of Anesthesiology, Henry Ford Health System, Detroit, MI USA; 4 Department of Anesthesiology and Critical Care, Hospital of the University of Pennsylvania, Philadelphia, PA USA; 5 Department of Anesthesiology and Perioperative Medicine, Oregon Health & Science University, Portland, OR USA; 6 Department of Anesthesia, Stanford University, Stanford, CA USA; 7 Department of Anesthesiology and Pain Medicine, University of Washington School of Medicine, Seattle, WA USA; 8 Department of Anesthesiology, Indiana University School of Medicine, Indianapolis, IN USA; 9 Department of Anesthesiology, Duke University School of Medicine, Durham, NC USA; 10 Department of Anesthesiology, University of Colorado Anschutz Medical Campus, Aurora, CO USA; 11 Department of Anesthesiology, College of Physicians & Surgeons of Columbia University, New York, NY USA; 12 Department of General Anesthesiology, Cleveland Clinic, Cleveland, OH USA

**Keywords:** Hypotension, Hypotension Prediction Index, Clinical decision support, Blood pressure monitor

## Abstract

**Background:**

Intraoperative hypotension is common during noncardiac surgery and is associated with postoperative myocardial infarction, acute kidney injury, stroke, and severe infection. The Hypotension Prediction Index software is an algorithm based on arterial waveform analysis that alerts clinicians of the patient’s likelihood of experiencing a future hypotensive event, defined as mean arterial pressure < 65 mmHg for at least 1 min.

**Methods:**

Two analyses included (1) a prospective, single-arm trial, with continuous blood pressure measurements from study monitors, compared to a historical comparison cohort. (2) A post hoc analysis of a subset of trial participants versus a propensity score-weighted contemporaneous comparison group, using external data from the Multicenter Perioperative Outcomes Group (MPOG). The trial included 485 subjects in 11 sites; 406 were in the final effectiveness analysis. The post hoc analysis included 457 trial participants and 15,796 comparison patients. Patients were eligible if aged 18 years or older, American Society of Anesthesiologists (ASA) physical status 3 or 4, and scheduled for moderate- to high-risk noncardiac surgery expected to last at least 3 h. Measurements: minutes of mean arterial pressure (MAP) below 65 mmHg and area under MAP < 65 mmHg.

**Results:**

Analysis 1: Trial subjects (*n* = 406) experienced a mean of 9 ± 13 min of MAP below 65 mmHg, compared with the MPOG historical control mean of 25 ± 41 min, a 65% reduction (*p* < 0.001). Subjects with at least one episode of hypotension (*n* = 293) had a mean of 12 ± 14 min of MAP below 65 mmHg compared with the MPOG historical control mean of 28 ± 43 min, a 58% reduction (*p*< 0.001). Analysis 2: In the post hoc inverse probability treatment weighting model, patients in the trial demonstrated a 35% reduction in minutes of hypotension compared to a contemporaneous comparison group [exponentiated coefficient: − 0.35 (95%CI − 0.43, − 0.27); *p* < 0.001].

**Conclusions:**

The use of prediction software for blood pressure management was associated with a clinically meaningful reduction in the duration of intraoperative hypotension. Further studies must investigate whether predictive algorithms to prevent hypotension can reduce adverse outcomes.

**Trial registration:**

Clinical trial number: NCT03805217. Registry URL: https://clinicaltrials.gov/ct2/show/NCT03805217. Principal investigator: Xiaodong Bao, MD, PhD. Date of registration: January 15, 2019.

**Supplementary Information:**

The online version contains supplementary material available at 10.1186/s13741-024-00369-9.

## Background

A constant supply of oxygen and nutrients is needed to support cellular metabolism. Adequate blood flow, blood pressure, and autoregulation help maintain organ perfusion. However, only blood pressure measurement is universally available and is a key component affecting clinical decision-making to ensure optimal organ perfusion. During noncardiac surgery, blood pressure is routinely measured intermittently every 3–5 min (American Society of Anesthesiologists, n.d.). Nevertheless, even with continuous monitoring of arterial blood pressure, intraoperative hypotension is common, with the incidence varying from 5 to 90%, depending on the chosen definition and the context (Bijker et al., 2007). For example, 20% of patients have at least one episode of mean arterial blood pressure (MAP) less than 65 mmHg during noncardiac surgery, and the incidence is over 88% in moderate- to high-risk patients (Gregory et al., 2021; Shah et al., 2020). Intraoperative hypotension is associated with perioperative morbidity and mortality, including acute kidney injury (AKI), myocardial injury, and stroke (Hallqvist et al., 2021; Salmasi et al., 2017; Sessler et al., 2018; van Waes et al., 2016; Wesselink et al., 2018; Wijnberge et al., 2021a). In addition, intraoperative hypotension increases hospital costs (Keuffel et al., 2019; Nanji et al., 2021). However, reactive clinical management delays the delivery of corrective intervention, making intraoperative hypotension difficult to prevent.

The AcumenTM Hypotension Prediction Index algorithm uses arterial pressure waveform information to alert clinicians of the patient’s likelihood of experiencing a future hypotensive event, defined as MAP < 65 mmHg for at least 1 min (Hatib et al., 2018). This proactive management may help clinicians treat hypotension effectively. The algorithm performance is validated in several small and mostly single-center studies (Hatib et al., 2018; Davies et al., 2020; Frassanito et al., 2022a; Frassanito et al., 2022b; Maheshwari et al., 2021; Ranucci et al., 2019; Shin et al., 2021; van der Ven et al., 2022; Wijnberge et al., 2021b). We conducted a single-arm multicenter prospective trial to evaluate the safety and effectiveness of the hypotension prediction algorithm to decrease hypotension in moderate- and high-risk noncardiac surgery patients, as compared to a historical comparison group. We hypothesized that the alert function of the prediction software would reduce the duration of hypotension, defined as minutes of MAP below 65 mmHg, by a clinically relevant amount. We also conducted a post hoc analysis to assess the duration of hypotension and incidence of AKI in patients who participated in the trial versus a propensity-score-weighted contemporaneous comparison group.

## Methods

### Trial design and ethics

The single-arm, prospective multi-center study was a post-market clinical study to further assess the safety and effectiveness of HPI. The trial was approved by a central Institutional Review Board (IRB; Western IRB approval #1-1131056-1) and 8 local IRBs. It was registered with clinicaltrials.gov (NCT03805217, registered 1/15/19, first participant enrolled 5/16/19, PI: Bao). Written informed consent was obtained from each subject in the prospective trial, but not from subjects who were retrospectively included in the comparison groups. Adverse events were reported by the trial sites, and an independent Clinical Events Committee reviewed event narratives, patient profiles, and hemodynamics to adjudicate all adverse events for attribution, severity, and relatedness to fluid management recommendations; classified as “not related”, “possibly related” or “related” to the software use, per FDA guidelines.

### Subject selection

Trial subjects were recruited from 11 academic hospitals across the USA, with no site exceeding 20% of the total enrollment. Three pilot subjects (pilot cohort) were permitted per site for training purposes before formal data acquisition began. We included adults ≥ 18 years old who were scheduled for elective moderate- or high-risk (defined by the primary anesthesia team), noncardiac surgical procedures, including orthopedic surgery, spine surgery, abdominal/pelvic surgery, or major peripheral vascular surgery, expected to last 3 h or longer. All subjects were American Society of Anesthesiologists (ASA) physical status 3 or 4 and required intra-operative mechanical ventilation and arterial catheterization for continuous blood pressure monitoring as part of their anesthetic care plan. Patients were excluded if they had significant cardiac valvular disease including aortic stenosis ≤ 1.5 cm^2^, moderate-to-severe mitral stenosis, moderate-to-severe aortic or mitral regurgitation; intra-cardiac shunt; atrial fibrillation; acute heart failure; on support by intra-aortic balloon pump or ventricle assist device or multiple vasoactive agents; sepsis; planned ventilation with tidal volume below 8 ml/kg of ideal body weight; as well as those scheduled for burn surgery, neurosurgical procedures, open-chest procedures or urgent/emergent surgery.

### Trial protocol

**Fig. 1:**
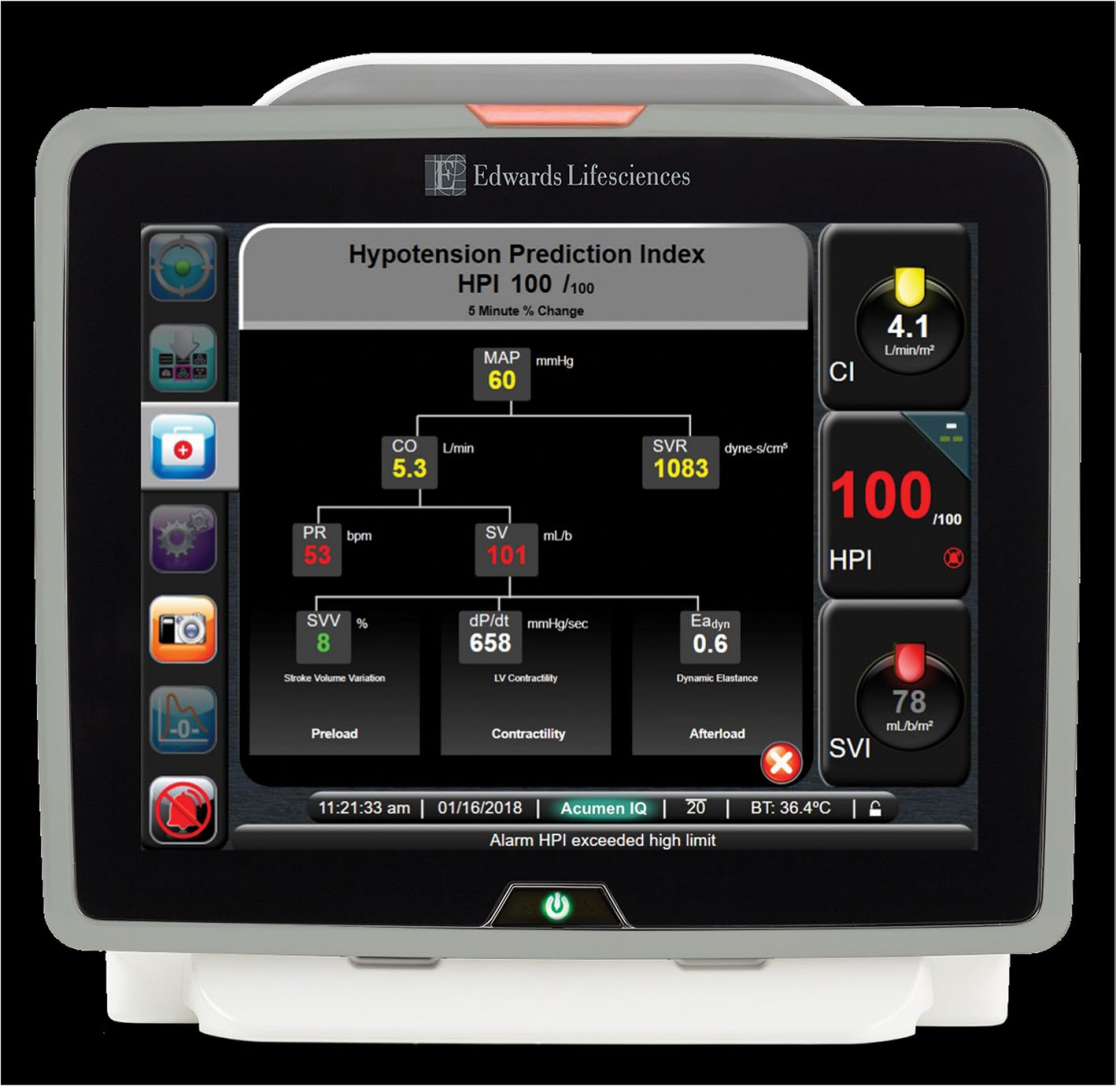
HPI Secondary Screen

Subjects were enrolled after signing informed consent and having an arterial catheter connected to FloTrac IQ sensor and EV1000 platform containing the predictive software. The software was activated after confirming a good-quality arterial waveform signal using a square wave test. The predictive index, ranging from 0 to 100, was displayed on the monitor, indicating the likelihood of patients having a hypotensive event. A secondary screen with quantitative hemodynamic parameters including cardiac output, stroke volume variation, change of pressure over the change of time, systemic vascular resistance, and dynamic arterial elastance aided clinician assessment of physiological conditions (Fig. 1). When the hypotension prediction index exceeded 85 for two consecutive 20-s updates, a popup alert appeared on the monitor to alert anesthesia providers. The care team could administer fluid and/or vasoactive agents using advanced hemodynamic data or could choose to ignore alerts based on their clinical assessment after coaching from the research team about the software.

### Trial endpoints

The primary endpoint for the trial was a cumulative duration of intraoperative hypotension. The secondary endpoint was the area under MAP of 65 mmHg. Data for the trial endpoints were downloaded from the EV1000 monitor, which records vitals and the predictive index every 20 s during the monitoring time. An episode of intraoperative hypotension was defined as three or more consecutive 20-s observations of MAP < 65 mmHg. Duration of hypotension was calculated as the sum of episodes where MAP < 65 mmHg for at least 1 min during the monitoring period. The area under MAP of 65 mmHg was calculated using the trapezoidal rule to estimate the area of pressure and time. The total area was obtained using the formula below.

$$\textrm{Total}\ \textrm{AUC}=\sum_1^k\sum_1^l\Big(\left({t}_{ij}-{t}_{\left(i-1\right)j}\right)\ast \left(65-\left(\frac{\left({p}_{ij}+{p}_{\left(i-1\right)j}\right)}{2}\right)\right)$$

where *t*_ij_ is the measurement time of the i_th_ hypotension increment of the *j*th hypotension episode for the patient and p_ij_ is the mean pressure in mmHg for the ith hypotension increment of the jth hypotension episode. The episode for each patient begins, *t*_0j_, with the first of two successive pressure measurements below MAP 65 mmHg and continues until the MAP raises to 65 mmHg or above. The trapezoidal rule sums the average decreases in pressure from 65 mmHg between two measurement times and multiplies that by the difference of the time increment between. Then, the areas per episode are summed across the total number of episodes.

### Statistical analysis

#### Single-arm trial with historical comparison group

A statistical analysis plan for the single-arm trial was written, date-stamped, and recorded in the investigators’ files before data were accessed (Appendix 1). The analysis excluded subjects in the pilot cohort. Mean duration of hypotension was calculated with a weighted average of site means and standard deviations as described in Appendix 1. The standard deviation of the duration of hypotension is the square root of the pooled variance with each study site’s hypotension variance. The trial participants were compared to a historical comparison cohort identified in registry data using t-tests. The Multicenter Perioperative Outcomes Group (MPOG) provided summary statistics on 22,109 adult patients with ASA 3 and 4 physical status, undergoing surgeries ≥ 180 min, with arterial line monitoring, treated between January 1, 2017 and December 31, 2017, at the same 11 hospitals that participated in the HPI effectiveness study (Shah et al., 2020). This information was used as a historical control to define the retrospective amount of IOH, which was compared to that found in this prospective HPI multicenter study. All trial analyses were completed with SAS version 9.4.

#### Post-hoc analysis with propensity score-weighted comparison group

To supplement the trial analysis that was designed *a priori*, we conducted a post hoc analysis that compared the duration of hypotension and the secondary outcome (AKI) among a subset of patients in the trial treatment group with a propensity-score weighted contemporaneous control group, using data reported from MPOG for hospitals that participated in the trial. Trial subjects, including both pilot and non-pilot subjects, from 10 of the 11 sites (“Trial”) were compared with a contemporaneous cohort of patients who had surgery in the same 10 sites from May 2019 to October 2020 and who did not participate in the trial (“Comparison”). Data were not available from MPOG for the 11th trial site for that time period. To maintain the inclusion and exclusion criteria from the trial, similar eligibility criteria were applied using definitions that could be applied to retrospective data, as specified in Appendix 2.

This post hoc analysis was conducted in collaboration with the MPOG consortium. The MPOG Site Primary Investigators at the HPI participating sites approved the use of the dataset for this project, and the analytic plan was presented at the MPOG Perioperative Clinical Research Committee. As has been previously described, the MPOG consortium (see www.mpog.org) maintains a detailed clinical and administrative data repository from participating hospitals across the United States. MPOG data include automated extraction of both device-captured and manually entered Electronic Health Record (EHR) data, including patient and procedural characteristics, anesthetic medications, physiologic parameters, and key surgical events for patients undergoing anesthesia care at contributing institutions. Monthly site-specific case validation for a random sample of submitted data by subject-matter experts is required of all contributing sites, and additional quality checks are conducted at the coordinating center to monitor each center’s data uploads and to remove artifacts from machine-captured variables.

Data elements from MPOG used in this analysis included patient demographics (age, sex, BMI, ASA status), clinical characteristics (Elixhauser comorbidities), procedural characteristics (procedure codes, blood pressure observations, vasopressor use, estimated blood loss), and patient outcomes (AKI).

The main outcome of the post hoc analysis was the duration of hypotension in minutes (defined as MAP < 65 mmHg for at least 1 min). The secondary outcome was AKI (using the Kidney Disease–Improving Global Outcomes definition of Stage 1 or greater, as an increase of serum creatinine more than 0.3 mg/dl above baseline within 48 h of anesthesia end time or more than 50% elevation within 7 postoperative days) (Disease, 2012).

A statistical analysis plan for the post hoc analysis, specifying the outcomes and methods, was drafted after the completion of the trial but before the post hoc analyses began (Appendix 2). Descriptive statistics for continuous data were reported as mean ± standard deviation or median (interquartile range) depending on the distribution of the data. Categorical data is presented as frequency counts and proportions. Weighted and unweighted standardized mean differences were calculated and reported to compare trial participants to the comparison group.

The post hoc analysis evaluated the association between the prediction software and the incidence of intraoperative hypotension, as defined above. To evaluate the difference in hypotension duration conditional on the use of the prediction software, a generalized linear model was conducted regressing the duration of hypotension on the fixed effects for software presence or absence. Given the skewed distribution for the duration of hypotension, a log link was specified to accommodate the distribution under study. Because it was anticipated that patients with longer surgical cases may have an increased period at risk of developing hypotension, the model was adjusted *a priori* for the duration of intraoperative blood pressure measurement (i.e., time at risk).

It was also anticipated that patients may have a different probability of participating in the trial, therefore the analysis employed the use of propensity score analyses. Specifically, individuals who elected to participate in the single-arm trial might possess characteristics that differentiate them from eligible individuals who either declined to participate or were not offered participation. To address this selection mechanism, a logistic regression model was first developed that predicts trial participation (i.e., yes vs no) conditional on demographic and disease characteristics. The predicted probability of participation was then used as an inverse probability of treatment weight (IPTW) in a second and final model (the primary analysis) that examines each outcome conditional on prediction software use (i.e., yes or no) and duration of blood pressure measurement. Variables in this IPTW included age, sex, race, ethnicity, ASA physical status, Vanwalraven Elixhauser Comorbidity Index, procedure timing (afternoon or morning), and procedural service. Several versions of the inverse-probability of treatment weights were considered but non-truncated and non-stabilized weights were chosen based on the distribution of estimated propensity scores. Results are presented as exponentiated coefficients that yield a percent difference (i.e., a ratio of geometric means) in the duration of hypotension between the trial and comparison groups and their associated 95% confidence intervals (CI).

The secondary outcome was evaluated using a similar approach but with a generalized linear model that included a binomial distribution and logit link function. Results of the secondary outcome are thus presented as an odds ratio and 95% confidence interval (CI). A sensitivity analysis was performed for both the primary and secondary outcomes using multiple imputation with chained equations (*m* = 40 imputations) which were derived from the preoperative clinical characteristics used in the propensity model.

All post hoc analyses were performed using R version 4.1.2 (R Foundation for Statistical Computing, Vienna, Austria) and RStudio (RStudio PBC, Boston, MA, USA), with two-sided *p* values < 0.05 considered statistically significant.

### Trial sample size estimate

Recent analyses demonstrated a mean duration of hypotension of 29.27 min with a standard deviation 43.44 (Shah et al., 2020). We anticipated that the use of the predictive algorithm would reduce that duration by 25%, based on based on an advisory panel expert opinion and review of recent literature (van Waes et al., 2016; Maheshwari et al., 2018; Stapelfeldt et al., 2017). The ratio of the standard deviation to the mean in the previous data was 1.44; however, the ratio from other publications on hypotension and data gathered by the sponsor varied between 0.88 to 1.76. To protect against the uncertainty of the ratio underpowering the study, additional computations were done to make the estimate more conservative with a standard deviation to mean ratio of 1.65. The minimum recommended required sample size, using Pass 14, is 380 completed subjects for 90% power for a one-sided alpha = 0.025 test. Assuming 10% attrition for a less than 3-h surgery and a 5% loss to follow-up, the recruited sample size was estimated as a minimum of 380/0.85 ≈ 448 for 90% power in the non-pilot cohort. Therefore, the study sample size was capped at 485, including up to 33 pilot subjects and up to 452 non-pilot subjects, for a minimum of 90% power.

## Results

### Single-arm trial with historical comparison group

A total of 778 patients were screened for trial eligibility (Supplementary Figure S1). Among them, 293 failed to meet inclusion criteria. Four hundred eighty-five subjects have consented to the study, 425 of whom ultimately had a surgery length of 3 h or longer (19 in the pilot cohort and 406 in the non-pilot cohort). The analysis focused on the 406 subjects in the non-pilot cohort and 293 of those who had at least 1 min of MAP below 65 mmHg. Table 1 shows the primary and secondary endpoints from the single-arm trial. During the study period, participants (*n* = 406) experienced a mean of 9 min (SD 13) of MAP < 65 mmHg, as reported via the EV 1000 monitors, and a mean area under MAP < 65 mmHg of 47 mmHg × minutes (SD 85). A historical comparison group from the same set of hospitals (*n =* 22,109) experienced a mean of 25 min (SD 41) of MAP < 65 mmHg, based on data from the MPOG registry, representing 65% fewer minutes of hypotension than trial participants (*p* < 0.0001). Subjects with at least one episode of hypotension (*n* = 293) had a mean of 12 ± 14 min of MAP below 65 mmHg compared with the MPOG historical control mean of 28 ± 43 min, a 58% reduction (*p* < 0.001). Trial sites reported a total of 21 postoperative safety events, including 17 incidents of AKI, 3 instances of myocardial injury, and 1 non-fatal cardiac arrest. No strokes or in-hospital deaths were observed.

**Table 1:** Hypotension outcomes from the single-arm trial

	Effectiveness including subjects with no episodes of MAP < 65 mmHg		Effectiveness among subjects with at least 1 min of MAP < 65 mmHg	
	Trial participants (*n* = 406)	Historical comparison cohort (*n* = 22,109)	Trial participants (*n* = 293)	Historical comparison cohort (*n* = 19,446)
	Mean (SD)	Mean (SD)	Mean (SD)	Mean (SD)
Trial primary outcome				
Duration of intraoperative hypotension (minutes of MAP < 65 mmHg)	9 (13)*	25 (41)	12 (14)*	28 (43)
Trial secondary outcome				
Area under MAP of 65 mmHg (mmH × minute)	47 (85)	na	65 (94)	na

*p < 0.0001. *MAP* mean arterial pressure, *na* not available, *SD* standard deviation

### Post hoc analysis with propensity score-weighted comparison group

**Fig. 2:**
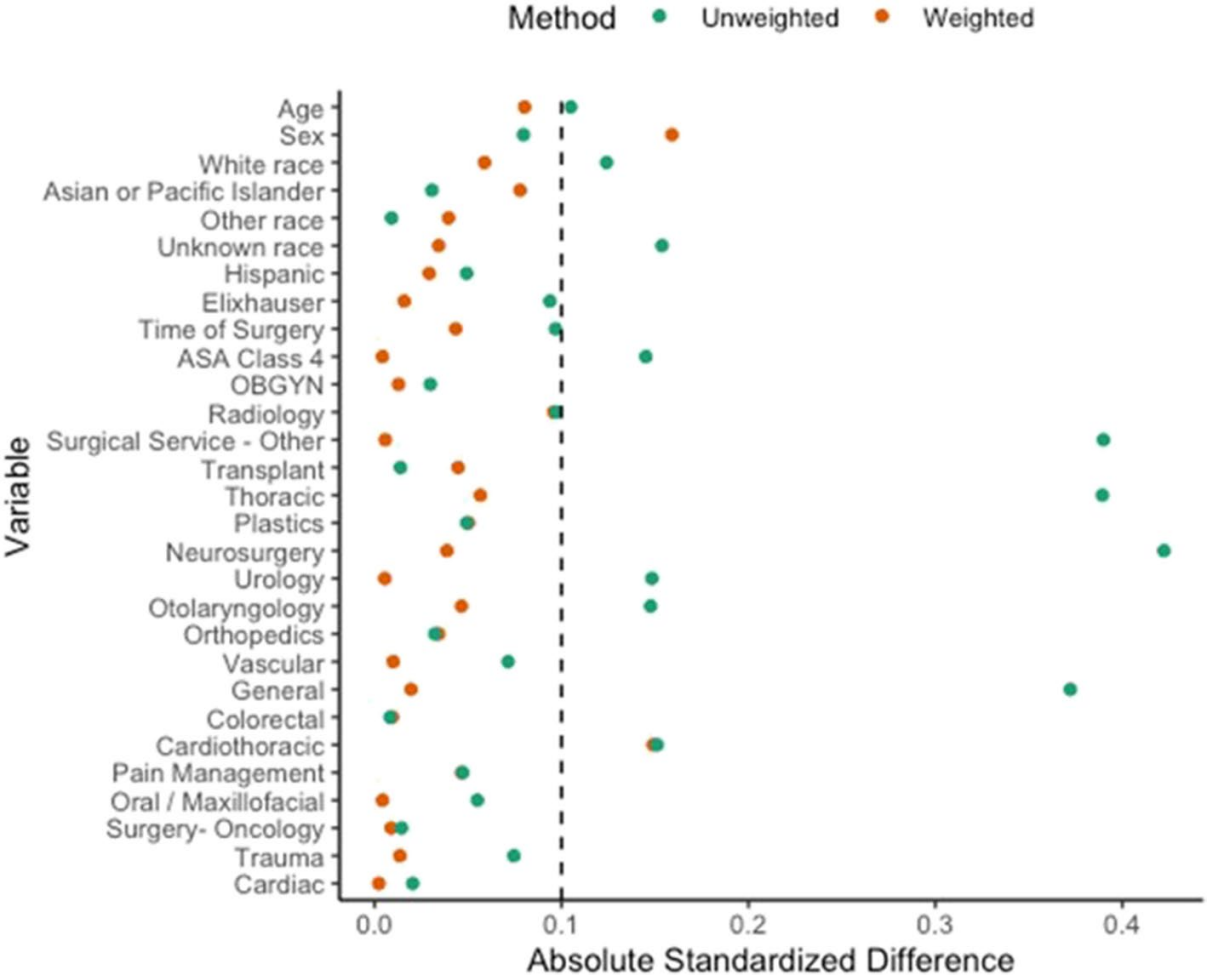
Balance Plot

The post hoc analysis focused on 457 subjects (pilot and non-pilot) from 10 of the 11 sites with the intention to treat the analysis for which data were available. MPOG identified in their registry 177,519 surgical cases from 10 trial sites that occurred during the trial recruitment period or within approximately 7 months thereafter. These were then limited to the 16,253 cases that matched the trial inclusion/exclusion criteria as closely as possible: noncardiac inpatient surgeries that lasted at least 3 hours and had hemodynamic monitoring through an arterial line (Supplementary Figure S2). Of those, 457 were participants enrolled in the trial, and the remaining 15,796 were designated as the comparison group. Table 2 shows the patient characteristics and surgical characteristics and the standardized mean difference between groups. Figure 2 shows a balance plot for the group characteristics, unweighted and weighted; it indicates that all but one of the weighted standardized mean differences between the trial and comparison groups are less than 0.1, suggesting that the groups are comparable on measured characteristics.

**Table 2:** Patient and surgical characteristics in the single-arm trial and contemporaneous comparison group

	Contemporaneous comparison *N = 15,796*	Trial *N = 457*	Standardized mean difference
Age, *years*	64.0 [54.0, 72.0]	65.0 [56.0, 72.0]	0.107
Male Sex^a^	8838 (56.0)	237 (51.9)	0.083
Race			0.172
White	12192 (77.2)	377 (82.5)	
Black	1417 (9.0)	37 (8.1)	
Asian or Pacific Islander	698 (4.4)	18 (3.9)	
Other	131 (0.8)	4 (0.9)	
Unknown	1358 (8.6)	21 (4.6)	
Hispanic or Latino ethnicity	314 (2.0)	6 (1.3)	0.053
ASA Physical Status Class IV	2186 (13.8)	40 (8.8)	0.161
Vanwalraven Elixhauser Comorbidity Index	11.0 [4.0, 19.0]	11.0 [3.0, 17.0]	0.094
AIDS/HIV^b^	47 (0.3)	1 (0.2)	0.017
Alcohol abuse^b^	294 (1.9)	4 (0.9)	0.085
Blood loss anemia^b^	378 (2.4)	7 (1.5)	0.063
Cardiac arrhythmias^b^	5451 (34.5)	108 (23.6)	0.244
Chronic pulmonary disease^b^	3606 (22.8)	87 (19.0)	0.095
Congestive heart failure^c^	1744 (11.0)	33 (7.2)	0.134
Coagulopathy^d^	1900 (12.0)	32 (7.0)	0.173
Deficiency anemia^d^	689 (4.4)	15 (3.3)	0.058
Depression^d^	2964 (18.8)	86 (18.8)	0.010
Diabetes complicated^c^	1382 (8.7)	36 (7.9)	0.033
Diabetes uncomplicated^c^	2703 (17.1)	89 (19.5)	0.061
Drug abuse^d^	727 (4.6)	11 (2.4)	0.120
Fluid electrolyte disorders^d^	4978 (31.5)	116 (25.4)	0.138
Hypertension, complicated^d^	3008 (19.0)	74 (16.2)	0.077
Hypertension, uncomplicated^d^	8132 (51.5)	242 (53.0)	0.030
Hypothyroidism^d^	2388 (15.1)	66 (14.4)	0.022
Liver disease^d^	1696 (10.7)	65 (14.2)	0.106
Lymphoma^d^	225 (1.4)	8 (1.8)	0.028
Metastatic cancer^d^	3183 (20.2)	121 (26.5)	0.150
Obesity^d^	3631 (23.0)	117 (25.6)	0.061
Other neurologic disorders^d^	1953 (12.4)	24 (5.3)	0.254
Paralysis^d^	602 (3.8)	7 (1.5)	0.142
Peptic ulcer disease excluding bleeding^d^	258 (1.6)	7 (1.5)	0.013
Peripheral vascular disorders^d^	2709 (17.1)	58 (12.7)	0.127
Psychoses^d^	165 (1.0)	4 (0.9)	0.020
Pulmonary circulation disorders^d^	959 (6.1)	17 (3.7)	0.110
Renal failure^d^	2777 (17.6)	71 (15.5)	0.057
Rheumatoid arthritis/collagen vascular diseases^d^	654 (4.1)	15 (3.3)	0.047
Solid tumor without metastasis^d^	6578 (41.6)	209 (45.7)	0.083
Valvular disease^d^	1284 (8.1)	18 (3.9)	0.178
Weight loss^d^	2291 (14.5)	58 (12.7)	0.055
Procedure started in afternoon^d^	5204 (32.9)	133 (29.1)	0.105
Procedural service			0.856
General	1971 (12.5)	124 (27.1)	
Orthopedics	1955 (12.4)	61 (13.3)	
Urology	1136 (7.2)	56 (12.3)	
Neurosurgery	4168 (26.4)	47 (10.3)	
Vascular	1483 (9.4)	33 (7.2)	
Transplant	877 (5.6)	23 (5.0)	
Otolaryngology	1129 (7.1)	17 (3.7)	
Obstetrics and gynecology	353 (2.2)	8 (1.8)	
Oral/maxillofacial	95 (0.6)	5 (1.1)	
Plastics	181 (1.1)	4 (0.9)	
Cardiac	93 (0.6)	2 (0.4)	
Colorectal	41 (0.3)	1 (0.2)	
Surgery-oncology	25 (0.2)	1 (0.2)	
Trauma	115 (0.7)	1 (0.2)	
Thoracic	1192 (7.5)	1 (0.2)	
Radiology	74 (0.5)	0 (0.0)	
Cardiothoracic	185 (1.2)	0 (0.0)	
Pain management	20 (0.1)	0 (0.0)	
Surgical service-other	673 (4.3)	70 (15.3)	
Other	30 (0.2)	3 (0.7)	

*ASA* American Society of Anesthesiologists: *HIV* human immunodeficiency virus: *AIDS* acquired immunodeficiency syndrome

Data is presented as median [quartile 1, quartile 3] or *n* (%) depending on the variable type

^a^Missing for 1 patient in the comparison group

^b^Missing for 435 patients in the comparison group and 12 patients in the trial

^c^Missing for 437 patients in the comparison group and 12 patients in the trial

^d^Missing for 441 patients in the comparison group and 12 patients in the trial

^d^Missing for 1 patient in the trial

Patients in the study group experienced fewer minutes of intraoperative hypotension than the comparison group [median 9 (3, 20) vs. 15 (5, 39) min, *p* < 0.001], as determined using EHR data from the MPOG registry (Table 3). In the inverse probability treatment weighing propensity model, patients in the trial had reduced total time spent in hypotension [exponentiated coefficient − 0.35 (95%CI − 0.43, − 0.27); *p* < 0.001]. The use of predictive software in hemodynamic management was associated with a 35% reduction in the duration of hypotension. There was no statistically significant difference in AKI (p = 0.637) between the two groups; 13.8% of trial participants and 15.8% of comparison patients had AKI. All inferences were consistent across a series of sensitivity analyses that used different modeling techniques, including propensity adjustment, multiple imputation propensity adjustment, and a post hoc sensitivity analysis adjusted for patient sex (Table 4).

**Table 3:** Comparison of intraoperative hypotension and acute kidney injury in the single-arm trial versus contemporaneous comparison group

	Contemporaneous comparison *N = 15,796*	Trial *N = 457*	Crude model^b^		Inverse probability of treatment weighing propensity model	
			Effect Estimate^c^ *(95% CI)*	*P*-Value	Effect estimate^c^ *(95% CI)*	*P* value
Duration of Hypotension (MAP < 65)^a^	15 [5, 39]	9 [3, 20]	− 0.31 (− 0.39 to − 0.22)	< 0.001	− 0.35 (− 0.43 to − 0.27)	< 0.001
Acute kidney injury	1898/12,421 (15.3%)	45/325 (13.8%)	0.93 (0.67 to 1.27)	0.674	0.91 (0.63 to 1.33)	0.637

*CI* confidence interval: *MAP* mean arterial blood pressure)

Data is presented as median [quartile 1, quartile 3] in minutes, or *n* (%) depending on descriptive statistics

^a^Data is only available for 15,749 observations

^b^All models are adjusting for time in which blood pressure is measured (i.e., time at risk)

^**c**^Effect estimates for hypotension are reported as exponentiated beta coefficients, whereas effect estimates for acute kidney injury are reported as odds ratios

**Table 4:** Model effect estimates, sensitivity analyses

Model^a^	Effect estimate^b^ *(95% CI)*	*P* value
Duration of hypotension (MAP < 65)		
Crude	− 0.31 (− 0.39 to − 0.22)	< 0.001
Propensity adjusted	− 0.33 (− 0.41 to − 0.24)	< 0.001
Inverse probability of treatment weighting	− 0.35 (− 0.43 to − 0.27)	< 0.001
Multiple imputation propensity adjustment	− 0.33 (− 0.41 to − 0.25)	< 0.001
Post hoc sensitivity–adjusted for sex	− 0.35 (− 0.32 to − 0.27)	< 0.001
Acute kidney injury		
Crude	0.93 (0.67 to 1.27)	0.674
Propensity adjusted	0.83 (0.60 to 1.14)	0.270
Inverse probability of treatment weighting	0.91 (0.63 to 1.33)	0.637
Multiple imputation propensity adjustment	0.88 (0.65 to 1.20)	0.432
Post hoc sensitivity–adjusted for sex	0.90 (0.61 to 1.31)	0.571

*CI* confidence interval: *MAP* mean arterial blood pressure

^a^All models are adjusting for time in which blood pressure is measured (i.e., time at risk)

^**b**^Effect estimates for hypotension are reported as exponentiated beta coefficients (i.e., ratio of expected geometric means), whereas effect estimates for acute kidney injury are reported as odds ratios

## Discussion

The goal of predictive technology is to warn clinicians and prevent untoward events by timely intervention. We report that the use of the hypotension prediction algorithm was associated with a 35% reduction in the duration of intraoperative hypotension versus comparison patients in a propensity-weighed model. Our results align with several single-center trials that have tested this software device. In a small, randomized controlled trial in the Netherlands, Wijnberge et al. (2020) observed a statistically significant reduction in time-weighted average MAP < 65 mmHg (Wijnberge et al., 2020). Their secondary outcome (median minutes of MAP < 65 mmHg) was 8.0 min in the intervention group vs 32.7 min in the control group. This is similar to the median of 9 min of hypotension we observed in the post hoc analysis of our multicenter trial group; however, our U.S. comparison group had a much lower duration of hypotension (median 15 min) than this European comparison group. A trial in Greece showed a 28% reduction in time-weighted average MAP < 65 mmHg in the intervention group, although they also observed an increase in hypertension and increased use of phenylephrine in the intervention group (Tsoumpa et al., 2021). Five other randomized trials observed that the use of the predictive algorithm reduced intraoperative hypotension (Grundmann et al., 2021; Schneck et al., 2020; Murabito et al., 2022; Šribar et al., 2023; Lorente et al., 2023), although they found no difference in lab values, clinical outcomes (Šribar et al., 2023), or tissue oxygenation (Lorente et al., 2023). An observational study of the software device found that those in the HPI group had less hypotension, fewer postoperative complications, and lower length of stay (Solares et al., 2023), and a second observational study saw shorter ICU ventilation time among the HPI group, although no difference in AKI (Reddy et al., 2023).

Of the known trials of this predictive algorithm, only one U.S.-based pilot study showed no difference in hypotension in the intervention and control groups, with both groups having a time-weighted average MAP < 65 mmHg of 0.14 mmHg (Maheshwari et al., 2020). A subgroup analysis by the authors demonstrated that the time-weighted average MAP < 65 mmHg reduced to 0.06 mmHg in the subset of alerts where anesthesia providers followed the study protocol. In addition, the comparison group had a low incidence of hypotension compared to the European trials (about one-third of the time-weighted average in the Wijnberge et al trial), possibly indicating the practice difference between the USA and Europe.

Although the use of the predictive algorithm was associated with a reduction in the duration of intraoperative hypotension, we did not observe a statistically significant reduction in AKI, our secondary outcome for the post hoc analysis. The incidence of AKI was 13.8% in the trial participants versus 15.3% in comparison patients. This study was not powered to detect a reduction in AKI; rather, AKI was one component of the composite safety outcome in the single-arm trial, and it was a secondary outcome for the post hoc comparison group analysis. The effect of hypotension on AKI could be influenced by patient baseline comorbidities and procedure risks. Mathis et al. (2020) reported no association of increased risk of AKI across all blood pressure ranges in patients with low risk and a strong association in patients of the highest risk (Mathis et al., 2020). The acute profound intraoperative hypotension could be hard to prevent and result in more kidney injury. Our trial did allow the freedom to anesthesia providers to ignore the alerts from devices. Although we achieved a 35% reduction in hypotension time, it may not be enough to convey kidney protection. Also, operations only account for a small portion of patients’ hospital stays, and hypotension could occur while patients are in postoperative care units, intensive care units, and floors, which would not be prevented by this trial.

It is worth noting that the measures of hypotension duration in the prospective single-arm trial (in Table 1) and the post hoc analysis with registry data (in Table 3) are not identical. Data on the duration of hypotension for the trial participants were extracted directly from the EV1000 monitor every 20 s, while the post hoc analysis outcome was calculated using blood pressure data from the anesthesia record at 1 min intervals. The EV 1000 monitor was attached to patients after the arterial line was placed and disconnected at the end of operations, which may not count all data from the anesthesia record and could miss volatile blood pressure swing at induction and emergence phases. Importantly, however, in the post hoc analysis that compares trial participants to cotemporaneous control patients, the hypotension data source and calculation method are the same for both groups, which makes the comparison valid. Additionally, in the prospective single-arm trial, 17 cases of AKI were reported by sites as adverse events by using the AKIN definition, whereas in the post hoc analysis, 45 instances of AKI were identified with KDIGO diagnosis criteria. It should be recognized that as a safety measure, kidney function was followed until postoperative day 3 in the prospective trial, while the registry data used for post hoc analysis reported kidney function until 7 days after operation, which may explain the discrepancy.

This study has several limitations. The trial group was subject to the Hawthorne effect; providers, aware of the trial, may have been more inclined towards aggressive correction of hypotension. In the initial analysis, the trial outcomes were compared to a historical comparison cohort; however, the data source differed between groups. The blood pressure data for trial participants was pulled from the EV 1000 monitors, whereas for the historical comparison cohort, it came from a clinical registry. Additionally, because we did not receive patient-level data for the comparison cohort, we were unable to control for differences in the patient and procedure between the groups. There is also a difference in methodology to calculate hypotension time. These limitations were the impetus for the post hoc analysis. The design of the post hoc analysis, which compares trial participants to a propensity-weighted comparison group, is able to control for observable differences between groups. The weighted models may not fully control for differences between the trial participants and comparison patients on variables that are not observed or measured in the registry data. Also, as noted above, the trial was not designed or powered to assess AKI, which we used as a secondary outcome in our post hoc analysis.

Despite the limitations, this analysis is a valuable addition to the literature on the use of predictive algorithms to guide hemodynamic monitoring and prevention of intraoperative hypotension. It is the first multicenter study of this predictive algorithm with the largest sample size and adds to the existing single-center trials. Additionally, while the post hoc design cannot provide as strong a causal relationship as a randomized control trial, the observational design with propensity-score weighted models is a rigorous method for estimating impacts in situations when a randomized trial is not possible for ethical, practical, or financial reasons. Our IPTW model provided a well-balanced match to compare to trial participants. This analysis demonstrates that real-world data can be used in conjunction with trial data to advance the evidence base.

There is now a large body of observational evidence to show that intraoperative hypotension increases patient risks for adverse outcomes related to perfusion, and there is a consensus statement recommending that anesthesia providers maintaining systolic arterial pressure above 100mmHg and MAP above 60 to 70 mmHg to attempt to reduce patient risk (Sessler et al., 2019). Nevertheless, prevention and management of hypotension should be targeted to the underlying physiological changes of volume status, cardiac contractility, and vascular tone. Aggressive or inappropriate volume overload or overuse of vasopressors without accounting for hypovolemia could lead to worsening surgical outcomes. A recent multicenter study demonstrated an increased incidence of AKI with decreased administration of crystalloid, shorter duration of hypotension, and higher usage of vasopressor (Chiu et al., 2022; Shin et al., 2018). EV 1000 monitor could potentially provide a quick insight into the patient’s hemodynamic status. More research is needed on the appropriate treatment for intraoperative hypotension in order to maintain hemodynamic stability most effectively while minimizing overtreatment (Chiu et al., 2022; Shin et al., 2018). Trials are also needed to more rigorously determine whether the use of predictive algorithms to prevent hypotension can reduce organ system damage and other complications, such as AKI, myocardial injury, postoperative delirium, and mortality.

## Conclusion

The use of prediction software for blood pressure management was associated with a clinically meaningful reduction in the duration of intraoperative hypotension. Further studies must investigate whether predictive algorithms to prevent hypotension can reduce adverse patient outcomes.

### Supplementary Information

**Additional file 1:** **Appendix 1.** Statistical Analysis and Reporting Plan

**Additional file 2:** **Appendix 2.** Statistical analysis plan

**Additional file 3:** **Supplementary Figure S1.** HPI Trial Diagram

**Additional file 4:** **Supplementary Figure S2.** Data Flow Diagram

## Data Availability

*Data from the trial:* There is a sub-set of data that support the findings of this study that are available from Edwards Lifesciences, but restrictions apply to the availability of these data, which were used under license for the current study, and so are not publicly available. Data are however available from the authors upon reasonable request and with permission of Edwards Lifesciences. *Data from the comparison group:* The data that support the findings of this study are available from the Multicenter Perioperative Outcomes Group but restrictions apply to the availability of these data, which were used under license for the current study, and so are not publicly available. Data are however available from the authors upon reasonable request and with permission of the Multicenter Perioperative Outcomes Group.

## Abbreviations

AKI: Acute kidney injury
ASA: American Society of Anesthesiologist
BMI: Body mass index
CI: Confidence interval
EHR: Electronic Health Record
HPI: Hypotension Prediction Index
IOH: Intraoperative hypotension
IPTW: Inverse probability of treatment weighted
KDIGO: Kidney disease: improving global outcomes
MAP: Mean arterial pressure
MPOG: Multicenter Perioperative Outcomes Group
SD: Standard deviation
