# Supplementary material for: AcumenTM hypotension prediction index guidance for prevention and treatment of hypotension in noncardiac surgery: a prospective, single-arm, multicenter trial
Source: Perioper Med (Lond). 2024 Mar 4;13:13. doi: 10.1186/s13741-024-00369-9 (PMC10913612; doi:10.1186/s13741-024-00369-9)
Supplement: Supplementary file 1 — Additional file 1: Appendix 1. Statistical Analysis and Reporting Plan [file 13741_2024_369_MOESM1_ESM.pdf]

STATISTICAL REPORT AND ANALYSIS PLAN

Title: Prospective, Single-Arm, Open-Label, Multicenter Study of Hypotension Prevention and Treatment in Patients Receiving Arterial Pressure Monitoring With Acumen™ Hypotension Prediction Index Feature Software (Clinical Protocol 2018-15)

Study Device: Acumen™ HPI Feature Software

Sponsor: Edwards Lifesciences LLC, One Edwards Way, Irvine, CA 92614 USA

Author: Richard P. Chiacchierini, Ph.D.

Version: C

Date: February 19, 2020

R.P. Chiacchierini & Associates, 15825 Shady Grove Road (Suite 30), Rockville, MD 20850
Phone: (240) 683-3738; Fax: (240) 683-9236; E-mail: rpc@rpcaconsulting.com

Analysis Summary

Study Title: Prospective, Single-Arm, Open-Label, Multicenter Study of Hypotension Prevention and Treatment in Patients Receiving Arterial Pressure Monitoring With Acumen™ Hypotension Prediction Index Feature Software (Clinical Protocol 2018-15)

Primary Objective: To determine whether the use of the Acumen™ HPI Feature Software to guide intraoperative hemodynamic management in non-cardiac surgery reduces the duration of intraoperative hypotension (defined as MAP < 65 mmHg for at least 1 minute) as compared with a historic retrospective control group.

Primary Effectiveness Endpoint: Duration (t [min]) reduction of intraoperative hypotension (defined as MAP < 65 mmHg for at least 1 minute) as compared with a historic retrospective control group.

Primary Safety Endpoints: Serious adverse events through 30 days as indicated below:

1. Serious Intraoperative and postoperative complications between each cohort;
2. Device-related serious adverse events.

Secondary Effectiveness Endpoint: The determination of total area under the curve (time and MAP) for IOH in each subject. This endpoint is correlated with the duration and a descriptive analysis of this endpoint will be presented with the mean, standard deviation, median, minimum and maximum.

Secondary Safety Endpoint: Percentages of subjects experiencing at least one of the following: postoperative episodes of non-fatal cardiac arrest, in-hospital death, stroke, AKI and MINS within 30 days of the procedure in the control and treated arms, respectively.

Additional Endpoints:.

1. Reduction of IOH duration at MAP ≤ 60 mmHg;
2. Area under threshold MAP < 65 mmHg
3. Acute kidney injury (AKIN criteria);
4. Quality of recovery (QoR-15) on postoperative day 3;
5. Postoperative morbidity survey (POMS) on postoperative day 3;
6. Amount of red blood cells infused (ml packed red blood cells);
7. Amount of intraoperative fluid (crystalloid and colloid);
8. Amount of vasoactive medications (i.e. phenylephrine, ephedrine, nor-epinephrine, (1)epinephrine, dobutamine);
9. Advanced hemodynamic variables at 20-sec intervals:
    a. Hypotension prediction index (HPI)
    b. Cardiac output (CO);
    c. Cardiac Index (CI);
    d. Stroke Volume (SV)
    e. Stroke Volume Index (SVI);
    f. Stroke Volume Variation (SVV);
    g. Dynamic arterial elastance (Eadyn);
    h. Maximal slope of the arterial pressure upstroke (dP/dt);
    i. MAP & PR
10. Hospital length of stay;
11. ICU Hospital length of stay
12. Hospital readmission within 30 days;
13. Cardiovascular mortality at 30 days;

14. Composite of systemic inflammatory response syndrome (SIRS) and at least one major organ dysfunction for renal, cardiovascular, respiratory, neurologic and coagulation systems by day 7 after surgery (2);
15. Acute respiratory distress syndrome (3);
16. Acute pulmonary edema (3);
17. Superficial or deep surgical site infection (3);
18. Hospital acquired infection (4);
19. Ventilator time.

Patient Population: Subjects recruited and enrolled should be classified as American Society of Anesthesiologist (ASA) physical status 3-4 who are scheduled for moderate-to-high-risk non-cardiac surgery and will be receiving pressure monitoring with an arterial line. Potentially qualifying patients will be evaluated during their preoperative anesthesia clinic visits. …

Study Design: A prospective, single-arm, open-label, multicenter post-approval study to determine whether the use of the Acumen™ HPI Feature Software to guide intraoperative hemodynamic management in non-cardiac surgery reduces the duration of intraoperative hypotension below a threshold of Mean Arterial Pressure (MAP).

Number of Patients: Up to 485 eligible subjects at up to 20 US study sites. The control group is comprised of 19446 patients. This number of controls is fixed and will not be altered.

Statistical Procedures

I. Introduction

Hemodynamic parameters that until recently were considered clinically acceptable, for instance, a MAP of 65 mmHg, are associated with both myocardial and renal injuries. At lower pressures, the association was stronger and only brief exposures were required. Associations based on relative thresholds were no stronger than those based on absolute thresholds. A strategy aimed at maintaining MAP above 65 mmHg appears to be as good as one based on the percentage reduction from baseline. (See figure 1) Absolute thresholds are easier to use in that they do not require a reliable baseline pressure and can thus more easily be incorporated into decision support systems (Salmasi et al. (2017)).

Multiple studies have also demonstrated that hypotensive duration is an important determinant of the likelihood, and possibly even the severity, of unfavorable outcomes (Meng et al. (2018)). Precise treatment of perioperative hypotension should be based on a reference to the patient's baseline measurements of BP, cardiac output, stroke volume, heart rate, and systemic vascular resistance. The execution of this proposal demands advanced hemodynamic monitoring that assesses volume and flow (Meng et al. (2018)).

The effects of intra-operative hypotension (IOH) on postoperative safety is a topic of great clinical concern. The association of IOH with postoperative death or substantial morbidity within 30 days has prompted clinicians to attempt to control IOH. The impact of this clinical concern is to question the ethics of having a concurrent untreated control group for any therapeutic agent to reduce the duration of IOH in non-cardiac surgical procedures. As a result of this concern, Edwards Life Sciences has devised a method to use a control population taken from a managed registry with patient-level data on non-cardiac surgical procedures. The registry described below provides the IOH duration of at least one minute duration for a recent time period expected to be unaffected by implementation of therapeutic methods to reduce the impact of IOH.

II. Study Design

This study is a prospective single-arm open-label multi-center post-approval study to determine whether the use of the Acumen™ HPI Feature Software to guide intraoperative hemodynamic management in non-cardiac surgery reduces the duration of intraoperative hypotension below a threshold of Mean Arterial Pressure (MAP). The comparison group for this study is a retrospective historical control group with patient-level data from the Multicenter Perioperative Outcomes Group (MPOG). The intention is to conduct the active therapeutic study at clinical centers enrolled in MPOG with HPI study patients being selected from the same centers. The methods described below are intended to provide a comprehensive analysis of the safety and effectiveness of the study data.

III. Analysis Objectives

The Primary Objective is to determine whether the use of the Acumen™ HPI Feature Software to guide intraoperative hemodynamic management in non-cardiac surgery reduces the duration of intraoperative hypotension (defined as MAP < 65 mmHg for at least 1 minute) as compared with a historic retrospective control group.

IV. Analysis Populations

A. Intent-to-Treat (ITT) Population

All subjects enrolled in the study comprises the intention-to-treat population. A subject will be considered enrolled in the study once the subject has signed the informed consent, has been assigned a Study Identification Number (SID), an arterial line has been placed and FloTrac IQ has been connected.

B. Full Analysis Set (FAS) Population

All enrolled subjects who undergo surgery of at least 3 hours duration and have one postoperative assessment comprises the full analysis set.

C. Completed (Evaluable) Cases (CC) Population

All patients who have had at least a 3-hour surgery and complete the 30-day follow-up comprises the completed cases population.

D. Per Protocol (PP) Population

All completed case subjects who do not have a protocol violation that could affect the primary endpoints of the study comprise the completed cases population.

V. Definition of Study Outcomes

A. Primary Effectiveness Variable

The primary effectiveness endpoint is the number of minutes of intraoperative hypotension (IOH) which is defined as a mean arterial pressure (MAP) below 65 for at least one minute experienced by patients undergoing elective non-cardiac surgery with ASA classifications of 3 or 4. It is anticipated that the alert function of the Acumen™ HPI Feature Software will reduce the duration of IOH by a clinically relevant amount. The null and alternative hypotheses for this endpoint are presented below.

H0: $\mu_T \geq \mu_C$ versus Ha: $\mu_T < \mu_C$

Where μ_T is the mean number of minutes of IOH for the HPI study subjects and μ_C is the mean not treated for IO derived from the historical control subjects. The FAS is the population in which the primary effectiveness endpoint will be tested.

1. Sample Size Computation

Recent analysis from the Multicenter Perioperative Outcome Group (MPOG) evaluated the mean duration of IOH in a subset of participating sites, during calendar year 2017, who met the eligibility criteria of this HPI Study. The analysis resulted in a total of 19446 patients with at least one minute of IOH and demonstrated a mean duration of IOH of 28.2 minutes with standard deviation 42.5.

It is anticipated that the HPI system will reduce that number of minutes by about 25%. Because the size of the standard deviation (SD) of the minutes in IOH decreases with decreasing mean. Estimates from other literature suggest that the SD is a similar fraction of the mean within the range. For this computation we assume that the ratio of the standard deviation to the mean remains constant. The ratio of the standard deviation of the mean from these data is 42.5/28.2 = 1.50. To protect against the uncertainty of the ratio under powering the study, additional computations were done to make the estimate more conservative with a SD to mean ratio of 1.65. The minimum recommended required sample size, using Pass 14, of 389 completed subjects for 90% power for a one-sided alpha = 0.025 test which were derived from the additional computations of standard deviation ratios. Assuming an attrition for a less than 3-hour surgery of 10% and a small loss to follow-up of 4%, the recruited sample size should be 389/0.86≈452 for 90% power in the Pivotal cohort. Therefore, the sample size for this study will be capped at 485, including up to 33 roll-in subjects and up to 452 Pivotal subjects, for a minimum of 90% power. Roll-in subjects will be analyzed separately from Pivotal subjects.

B. Primary Safety Variable

The primary safety endpoint is the percentage of serious adverse events to include perioperative events, postoperative complications, and device-related serious adverse events.

C. Secondary Effectiveness Endpoints

The secondary effectiveness endpoint is the determination total area under the curve of the time and MAP for all time periods for which MAP < 65 mmHG in each subject.

D. Secondary Safety Endpoints

The secondary safety endpoint is a composite of the following events within 30 days of the procedure: postoperative non-fatal cardiac arrest, in-hospital death, stroke, acute kidney injury (AKI), and myocardial injury in non-cardiac surgery.

E. Additional Endpoints

2. Reduction of IOH duration at MAP ≤ 60 mmHg.

The duration of IOH considering a lower cut point will be estimated for the study subjects.

3. Area under threshold MAP < 65 mmHg

A measure highly correlated with duration of IOH is the area under the threshold of 65 mmHg. The area is computed by using the trapezoidal rule to estimate the area of pressure and time. The Total AUC is obtained using the formula below.

$$Total\ AUC = \sum_1^k \sum_1^l ((t_{ij} - t_{(i-1)j}) * (65 - (\frac{(p_{ij} + p_{(i-1)j})}{2}))$$

where t_{ij} is the measurement time of the ith IOH increment of the jth IOH episode for the patient and p_{ij} is the mean pressure in mmHG for the ith IOH increment of the jth IOH episode. The episode for each patient begins, t_{0j}, with the first of two successive pressure measurements below MAP 65 mmHG and continues until the MAP raises to 65 mmHG or above. The trapezoidal rule sums the averages the decreases in pressure from 65 mmHG between two measurement times and multiplies that by the difference of the time increment between. Then the areas per episode are summed across the total number of episodes.

4. Acute kidney injury (AKIN criteria).

The percentage of study subjects with acute kidney injury under the AKIN criteria will be estimated.

5. Quality of recovery (QoR-15) on postoperative day 3.

The scores of QoR-15 will be computed and reported.

6. Postoperative morbidity survey (POMS) on postoperative day 3.

The postoperative morbidity will be assessed with the POMS on the third day after surgery.

7. Amount of red blood cells infused (ml packed red blood cells).

The amount of transfused blood product will be estimated and reported for study subjects.

8. Amount of intraoperative fluid (crystalloid and colloid).

The volume of intraoperative fluids administered to study subjects will be reported.

9. Amount of vasoactive medications (i.e. phenylephrine, ephedrine, nor-epinephrine, (1) epinephrine, dobutamine).

The type and amount of vasoactive medication administered to study subjects will be reported.

10. Advanced hemodynamic variables at 20-sec intervals.

a. Hypotension prediction index (HPI).

A descriptive summary of the HPI will be provided for study subjects.

b. Cardiac output (CO).

A descriptive summary of cardiac output will be summarized and provided for study subjects.

c. Cardiac Index (CI).

A summary of the cardiac index summarized and will be reported for study subjects.

d. Stroke Volume (SV).

The stroke volume will be summarized and reported for study subjects.

e. Stroke Volume Index (SVI).

The stroke volume will be summarized and provided for study subjects.

f. Stroke Volume Variation (SVV).

The stoke volume variation will be summarized and reported for study subjects.

g. Dynamic arterial elastance (Eadyn).

The Eadyn will be summarized and presented for study subjects.

h. Maximal slope of the arterial pressure upstroke (dP/dt).

The dP/dt will be summarized and reported for study subjects.

i. MAP & PR .

The mean arterial pressure and pulse rate will be summarized and reported for study subjects.

11. Hospital length of stay.

The length of stay in the hospital will be summarized and reported for study subjects.

12. ICU Hospital length of stay.

The length of stay in ICU will be summarized and reported for study subjects.

13. Hospital readmission within 30 days.

The percentage of study subjects with readmission to the hospital will be provided with the reasons for readmission.

14. Cardiovascular mortality at 30 days.

The percentage of study subjects who day within 30 days will be presented.

15. Composite of systemic inflammatory response syndrome (SIRS) and at least one major organ dysfunction for renal, cardiovascular, respiratory, neurologic and coagulation systems by day 7 after surgery.

The percentage of study subjects with this composite will be reported.

16. Acute respiratory distress syndrome.

The percentage of study subjects with acute respiratory distress syndrome will be reported.

17. Acute pulmonary edema.

The percentage of study subjects with acute pulmonary edema syndrome will be reported.

18. Superficial or deep surgical site infection.

The percentage of study subjects with surgical site infection will be reported.

19. Hospital acquired infection.

The percentage of study subjects with hospital acquired infection will be reported.

20. Ventilator time.

The ventilator time of study subjects will be summarized and presented.

VI. Multiplicity

The testing for the primary effectiveness analysis will be a one-sided 0.025 test. The primary effectiveness analysis must meet its objective of statistical significance. After analysis of the primary effectiveness endpoint, if the primary analysis results in statistical significance, the descriptive secondary effectiveness endpoints will be presented with nominal two-sided 95% confidence intervals. Thus, the study will be protected from alpha inflation by the closed form hierarchical method. Multivariate analyses are considered supportive without control of alpha and the additional endpoint analyses are likewise supportive without control of alpha.

VII. Data Pooling

Data from all study sites will be pooled for all analyses in this study based on the clinical criteria of Meinert (1986): all sites followed the same protocol, the sponsor monitored the sites for protocol compliance, and the data gathering mechanism was the same at each site. A sensitivity analysis will be done from the primary and secondary endpoints to determine of study site has an impact on the analysis endpoint.

VIII. Interim Analysis

There will not be an interim analysis done in this study.

IX. Statistical Analyses

A. Character of Study Variables

As a routine evaluation of the data during analysis, consistency of the study variables to properties of statistical tests will be done. For continuous variables, equality of variance tests will be done to support analyses requiring this condition such as the Wilcoxon rank sum test. With categorical data, exact statistical test procedures will be to minimize test assumptions. For tabulated continuous variables, the descriptive analyses will present the mean, standard deviation, median, minimum and maximum. For tabulated categorical variables, the number with the characteristic, the total number evaluated, the percent and the 95% exact binomial confidence intervals will be provided.

B. Comparability Analyses of the Patient Populations

These analyses are intended to determine the similarity of treatment groups and study sites with respect to important demographic or other variables, either known or suspected to have an influence on the outcome variables. The absence of similarity for any baseline variable will identify that variable as a potential covariate in subsequent safety and effectiveness multivariable analyses. The data for each baseline variable will be presented descriptively. For quantitative variables like age, the mean, standard deviation (SD), median, minimum, and maximum will be presented. For qualitative variables like gender, the number with the characteristic, the total number evaluated, the rate, and the exact 95% binomial confidence limits will be presented.

1. **Comparability between Treatments**

The comparison of baseline characteristics across treatment groups will apply the following methods. The quantitative variables such as age will be done with two-sided α=0.05 Wilcoxon rank sum test or two-sample t-test, and qualitative variables will be analyzed with Fisher's exact test or Fisher-Freeman-Halton test. Any variable found to have $P<0.10$ will be used as a possible covariate in subsequent multivariate safety or effectiveness analyses.

2. **Comparability between Study Sites**

An analysis of comparability across study sites without regard to treatment assignment will be carried out by the same baseline characteristics by the analysis methods described above. Study site differences do not disallow pooling, but variables including study site and the variable found different need to be considered as covariates in subsequent multivariate analyses.

C. Patient Accountability and Missing Data

A summary table will provide the total number of subjects enrolled and completed. The subjects eligible for and compliant with each follow-up contact will be summarized descriptively. Subjects withdrawn will be tabulated with their reasons for withdrawal.

Every effort will be made to collect all data points in the study. The sponsor plans to minimize the amount of missing data by appropriate management of the prospective clinical study, proper screening of study subjects, and training of participating investigators, monitors and study coordinators. The sponsor will provide a list of subjects who do not complete the trial along with the best information available on why they each left the trial prematurely.

While no imputation will be done for this study, sensitivity analysis by developing multivariate models of duration of IOH as a function of baseline and preoperative characteristics. If successful, the models may allow regression imputation for subjects with that are considered missing at random. Subjects with missing data that result from adverse events related to the test device or its use will be counted as not missing at random and will be imputed in the sensitivity analysis buy random selection of a value with the same mean and SD as the untreated control distribution. Missing at random subjects will be imputed by random selection from a distribution with the same mean and SD as the treated subjects who have complete data.

D. Primary Effectiveness

Recall that the study subjects were drawn from the same study sites that were used for the MPOG estimates from the control population. Because the sites may represent different patient populations, it is likely that the estimates from the site may be heterogeneous as were the estimates for the control MPOG sample. In an ideal world the proportion of subjects in the study will be similar to that from the MPOG controls, but the dynamics of study conduct usually result in a different subject distribution depending on which study sites were entered into the study earlier rather than later. In order to provide an estimate from the study sample that is most comparable to the controls adjusting for site the primary effectiveness endpoint will be done with a weighted average of site means and standard deviations as described below.

The study subjects will have the mean and standard deviation of the durations of IOH during the study computed by study site. To generate an estimate across the range of estimates for comparison to the MPOG controls, the estimates from each study site will be weighted and combined in the same proportion of subjects that were included in the control sample from MPOG. This method is analogous to direct standardization used in epidemiology (Fleiss et al (2003)). This weighted average and its properly computed standard deviation will be compared to the estimates obtained from MPOG by a one -sided unequal variance two-sample t-test. If this test is statistically significant with $P<0.025$, the primary effectiveness endpoint will be a success. If the proportion of subjects in the

control sample is p_k for study site k and the estimate of the mean from study site k is $\bar{x}_k$, then the study estimate from the interim sample for the overall mean is given below.

$$\bar{x} = \sum_k p_k \bar{x}_k.$$

If the standard deviation for study site k is s_k, then the estimate of the interim standard deviation is given below.

$$s = \sqrt{\sum_k (p_k s_k)^2}.$$

The test statistic for the interim is taken from the formula below.

$$t = \frac{\bar{x} - \bar{x}_{MPOG}}{\sqrt{\frac{s^2}{n} + \frac{s^2_{MPOG}}{n_{MPOG}}}}.$$

There are several sensitivity analyses to be done after the completion of the primary analysis. Assuming de-identified patient level data is available from MPOG, a multivariable mixed models analysis of variance will be done to determine if there are relevant impacts of patient characteristics that may impact the primary endpoint. Factors of concern will include gender, age, race, BMI, prior history of hypotension, prior history of heart disease, prior history of smoking, angiotensin receptor blockers, calcium channel blockers and ace inhibitors, ASA status, baseline MAP, history of coagulopathy, surgical time, intra-operative blood loss, diabetes, development of sepsis as an AE, chronic pulmonary disease and any baseline characteristic found out of balance by treatment group or study site. Factors will initially be screened with models that include treatment, the factor of interest, and the interaction between the two. Because of the increased power of the control sample, many of these comparisons could be statistically significant but not clinically significant. The screened factors will be evaluated to determine which should be included in a competition for the final model. This analysis will be done by backward elimination with no factors remaining in the final model with P>0.05.

If it is not possible to get patient level data from MPOG, it may be possible for MPOG to perform the modeling by supplying them with the de-identified study data from our subjects.

From the model developed above, a second sensitivity analysis can be done to attempt to estimate the duration of IOH in subjects with missing data based on the model-based regression imputation (Little and Rubin, 2002). If a plausible model can be obtained, the imputation will be done with 10 different randomization seeds yielding ten populations from which a statistical test of the weighted site estimates can be used to provide supporting analysis to that done for the primary endpoint. The 10 imputed tests will be combined by the method of Rubin (1987) (this method is the substance of the SAS procedure MIANALYZE). The imputations will be done consistent with the guidance from the National Research Council Committee on National Statistics (2010).

Finally, a sensitivity analysis will be done on all patients regardless of the duration of surgery (including patients excluded from the primary endpoint analysis with less than three hours of surgery). The site averages and SDs will be combined by the method of weighting above and that mean and standard deviation will be compared to that from MPOG for all patients with at least 3 hours of surgery.

E. Primary Safety

The serious adverse events subdivided by perioperative, postoperative, and device related events will be presented descriptively. The number of subjects experiencing at least one event, the total number evaluated, the percentage, the exact 95% confidence interval on the percentage and total number of events will be presented

F. Secondary Effectiveness

The cumulative area under the curve will be computed by the trapezoidal rule with the MAP as the amplitude and the duration as the base. The area under the curve computation involves summing across all time points the average amplitude for the beginning and ending time point times the unit of time between the two time points summed across all times when MAP was <65. This endpoint is correlated with the duration and a descriptive analysis of this endpoint will be presented with the mean, standard deviation, median, minimum and maximum across all patients with IOH of at least one-minute duration.

G. Secondary Safety

The descriptive presentation will be given for the 30-day composite endpoint with any of the following events: postoperative non-fatal cardiac arrest, in-hospital death, stroke acute kidney injury, and myocardial injury in this non-cardiac surgery. This endpoint will be presented descriptively with the number of subjects experiencing one or more of the composite events, the total number evaluated, the percentage and the exact 95% confidence limits on the percentage. The number of subjects experiencing each element of the composite with the number of events will also be presented descriptively.

H. Additional Effectiveness Endpoint Analyses

For each additional endpoint listed above the presentation will be descriptive. For continuous endpoints, the mean, standard deviation, number evaluated, median, minimum, and maximum will be presented. For categorical endpoints, the number with the property, the total number evaluated, the percentage, and the exact 95% confidence limit on the percentage will be provided.

X. Statistical Software

The primary analyses will be done using SAS, Version 9.4 or later for Personal Computers. The Fisher's exact tests, exact 95% confidence limits, and other categorical data computations will be done with StatXact for windows (Version 8 or later). Some preliminary descriptive analyses and figures may be done with Minitab Version 17 or later.

XI. References:

1. Salmasi, M.D., S. Vafi, et al. (2017) Relationship between Intraoperative hypotension, defined by either reduction from baseline or absolute thresholds, and acute kidney and myocardial injury after noncardiac surgery. *Anesthesiology* (126): 47-65.
2. Meng, L., W. Yu, T. Wang, L. Zang, P. Heerdt, and A. Gelb. (2018). Blood pressure targets in perioperative care provisional considerations based on a comprehensive literature review. *Hypertension:*(72): 806-817.
3. Meinert, C, 1986. Clinical Trials: Design, Conduct, and Analysis. Oxford University Press, New York.
4. National Research Council. (2010). *The Prevention and Treatment of Missing Data in Clinical Trials.* Panel on Handling Missing Data in Clinical Trials. Committee on National Statistics, Division of Behavioral and Social Sciences and Education. Washington, DC: The National Academies Press.
5. Little, R and Rubin, D, 2002. Statistical Analysis with Missing Data. John Wiley and Sons, New York.
6. Rubin, D. (1987) *Multiple Imputation for Nonresponse in Surveys.* John Wiley and Sons, New York.

R.P. Chiacchierini & Associates, 15825 Shady Grove Road (Suite 30), Rockville, MD 20850
Phone: (240) 683-3738; Fax: (240) 683-9236; E-mail: rpc@rpcaconsulting.com

7. SAS OnlineDoc™ Version 9. *Combining Data from Imputed Data Sets*. The MIANALYZE Procedure. SAS, Cary, NC.

R.P. Chiacchierini & Associates, 15825 Shady Grove Road (Suite 30), Rockville, MD 20850
Phone: (240) 683-3738; Fax: (240) 683-9236; E-mail: rpc@rpcaconsulting.com
