# Supplementary material for: AcumenTM hypotension prediction index guidance for prevention and treatment of hypotension in noncardiac surgery: a prospective, single-arm, multicenter trial
Source: Perioper Med (Lond). 2024 Mar 4;13:13. doi: 10.1186/s13741-024-00369-9 (PMC10913612; doi:10.1186/s13741-024-00369-9)
Supplement: Supplementary file 2 — Additional file 2: Appendix 2. Statistical analysis plan [file 13741_2024_369_MOESM2_ESM.docx]

Statistical Analysis Plan

Prospective, Single-Arm, Open-Label, Multicenter Study of Hypotension Prevention and Treatment in Patients Receiving Arterial Pressure Monitoring With Acumen™ Hypotension Prediction Index Feature Software

*Multicenter Perioperative Outcomes Group (MPOG) PCRC-0061 Amendment*

Version 05/25/2021

# Administrative Information

| **Study Name** | Prospective, Single-Arm, Open-Label, Multicenter Study of Hypotension Prevention and Treatment in Patients Receiving Arterial Pressure Monitoring With Acumen™ Hypotension Prediction Index Feature Software |
| --- | --- |
| **Study Acronym** | MPOG HPI Study |
| **PCRC Number** | PCRC-0061 |
| **Principal Investigator** | Xiaodong Bao, MD PhD |
| **Study Statistician(s)** | Timothy T Houle, PhD  Ariel Mueller, MA  Kathryn Cody, MPH |
| **Study Sponsor** | Edwards Lifesciences |
| **Study Design** | Retrospective Cohort Study |
| **Associated Document(s)** | Clinical Protocol Version 4.0 (29-Jan-2020)  PCRC-0061 Amendment (15-Jun-2021) |

# Signature Page

I give my approval for the attached SAP entitled “Prospective, Single-Arm, Open-Label, Multicenter Study of Hypotension Prevention and Treatment in Patients Receiving Arterial Pressure Monitoring With Acumen™ Hypotension Prediction Index Feature Software – MPOG PCRC-0061 Amendment” dated 05/25/2021.

**Senior Statistician**

_________________________________ ___________________

*Timothy T Houle, PhD Date*

Contents

[Administrative Information 1](#_Toc107329259)

[Signature Page 2](#_Toc107329260)

[Summary of Amendments 4](#_Toc107329261)

[Introduction 5](#_Toc107329262)

[Scope of the Analysis 5](#_Toc107329263)

[Study Objectives 5](#_Toc107329264)

[Hypothesis and Objectives 5](#_Toc107329265)

[Primary Hypothesis 5](#_Toc107329266)

[Secondary Hypotheses 5](#_Toc107329267)

[Study Methods 6](#_Toc107329268)

[Data Sources 6](#_Toc107329269)

[Detailed Inclusion and Exclusion Criteria 6](#_Toc107329270)

[Study Exposures and Outcomes 7](#_Toc107329271)

[Exposure 7](#_Toc107329272)

[Outcomes 7](#_Toc107329273)

[General Analysis Considerations 8](#_Toc107329274)

[Statistical Principles 8](#_Toc107329275)

[Power Analysis 8](#_Toc107329276)

[Descriptive Statistics 8](#_Toc107329277)

[Primary Analysis 8](#_Toc107329278)

[Secondary Analyses 9](#_Toc107329279)

[Sensitivity and Exploratory Analyses 9](#_Toc107329280)

[Reporting Considerations 9](#_Toc107329281)

[Reporting Conventions 9](#_Toc107329282)

[Quality Assurance of Statistical Programming 9](#_Toc107329283)

[Statistical Software 9](#_Toc107329284)

[Role of the Study Sponsor 10](#_Toc107329285)

# Summary of Amendments

All amendments to the study protocol that update the analytical approach will be submitted for review and documented below. Further, any change to the proposed statistical methods, especially in relation to any underlying understanding about the study data will be documented below, including the timing and justification for the change.

| **Version** | **Date** | **Item / Section** | **Details** |
| --- | --- | --- | --- |
| V1 | 2021-05-25 | SAP / All Sections | Initial SAP Draft |
|  |  |  |  |
|  |  |  |  |
|  |  |  |  |
|  |  |  |  |
|  |  |  |  |

# Introduction

This study makes use of two existing research projects evaluating hypotension in the operative setting. Briefly, the use of the Acumen™ Hypotension Prediction Index Feature Software was evaluated in a prospective, single-arm clinical trial, denoted the HPI study. Rates of hypotension in this cohort were descriptively compared to patients in study by Nirav Shah *et al*, in which they assessed the association between prolonged intraoperative hypotension, acute kidney injury, and other adverse surgical outcomes using the Multicenter Perioperative Outcomes Group (MPOG) database. As an extension of the MPOG project, this study will include a formal inferential analysis to determine whether these rates varied among those who did and did not receive the hypotension prediction software at select MPOG institutions after accounting for important confounding influences. It is hypothesized that the HPI technology would reduce the duration of intraoperative hypotension and improve surgical patient outcomes as compared to the standard of care.

**Primary Objective**: To determine whether the use of the Acumen^TM^ HPI Feature Software to guide intraoperative hemodynamic management in non-cardiac surgery reduces the duration of intraoperative hypotension (defined as MAP < 65 mmHg for at least 1 minute) as compared to the standard of care.

**Secondary Objectives**: To determine if the guidance provided by the Acumen^TM^ HPI Feature Software reduces the incidence of acute kidney injury (AKI) within 30 days of the procedure, and postoperative nausea and vomiting (PONV) in the immediate postoperative period.

## Scope of the Analysis

This document describes the statistical analysis and provides instructions for statistical programming of the MPOG PCRC amendment, in reference to the study objectives outlined below. This includes assessment of the Acumen™ Hypotension Prediction Index Feature Software in the MPOG dataset using only data that is routinely collected as part of clinical care and uploaded to the MPOG database.

Details of the analysis for the prospective, single-arm, multicenter trial are described in a separate statistical analysis plan as described in the main clinical trial protocol.

# Study Objectives

The proposed project is aimed to extend the previous retrospective cohort study using contemporaneous controls to examine the effects of the HPI technology on hypotension and adverse clinical events following non-cardiac surgery using MPOG and HPI study data. This study has been reviewed and approved by the Institutional Review Board at Massachusetts General Hospital (Protocol 2019P001582) including this exploratory data analysis of MPOG data. Results will be reported in accordance with the STROBE and RECORD guidelines.

## Hypothesis and Objectives

### Primary Hypothesis

Null Hypothesis (H_0_): There is no difference in the mean duration of hypotension between patients who received HPI and those who did not receive the HPI technology.

Alternative Hypothesis (H_A_): There is a difference in the mean duration of hypotension between patients who received HPI and those who did not receive the HPI technology.

H_0_: μ_hpi_ = μ_nohpi_ vs H_A_: μ_hpi_ ≠ μ_nohpi_,
*_where μ represents the mean duration of hypotension_*

### Secondary Hypotheses

AKI Null Hypothesis (H_0_): There is no difference in the probability of AKI between patients who received HPI and those who did not receive the HPI technology.

AKI Alternative Hypothesis (H_A_): There is a difference in the probability of AKI between patients who received HPI and those who did not receive the HPI technology.

H_0_: P(AKI)_hpi_ = P(AKI)_nohpi_ vs H_A_: P(AKI)_hpi_ ≠ P(AKI)_nohpi_,

*_where P(AKI) represents the probability of developing AKI postoperatively_*

PONV Null Hypothesis (H_0_): There is no difference in the probability of PONV between patients who received HPI and those who did not receive the HPI technology.

PONV Alternative Hypothesis (H_A_): There is a difference in the probability of PONV between patients who received HPI and those who did not receive the HPI technology.

H_0_: P(PONV)_hpi_ = P(PONV)_nohpi_ vs H_A_: P(PONV)_hpi_ ≠ P(PONV)_nohpi_*,*

*_where P(AKI) represents the probability of developing AKI postoperatively_*

# Study Methods

## Data Sources

This data will utilize the Multicenter Perioperative Outcomes Group (MPOG) database for analysis. Briefly, the MPOG database is based off a collaborative network of hospitals that have automated data abstraction from their electronic health record in order to participate in multicenter anesthesia research, education and quality improvement. Specific details regarding the MPOG database, and its procedures for data validation and quality control are available on their website (<https://mpog.org/>).

For this study, each participating HPI institution will identify their local MPOG Case ID for patients that consented to participate in the prospective HPI study. The list of MPOG Case IDs will be sent directly from each site to the MPOG Coordinating Center. These IDs will then be linked to the MPOG database to create a binary indicator for all patients that indicates whether that case used the HPI technology. This linkage will allow investigators at MGH to analyze the proposed research question without being able to identify specific patients from other institutions.

## Detailed Inclusion and Exclusion Criteria

This study involves assessment of non-cardiac surgical cases that closely mirrors the inclusion criteria of the parent prospective trial. Therefore, all patients included in the prospective parent trial will be included. Control cases (not receiving the HPI technology) will be included only from hospitals that participated in the HPI prospective study and agreed to participate in this retrospective cohort study. Consistent with the previous query, a contemporaneous cohort of patients presenting between 05/2019 and 02/2020 will be included as controls if they did not receive the HPI technology. Specific inclusion and exclusion criteria for control cases are outlined below:

**Inclusion Criteria**

- Adult patients (greater than 18 years of age)
- Non-cardiac cases, *defined using cases in which the MPOG Concept 80005 for cardiac cases is not present*
- Elective same day admission or inpatient
- Surgical time ≥ 3 hours
- Patients with a documented arterial line

**Exclusion Criteria**

- ASA 5 or 6
- Emergency case
- Outpatient case
- Undergoing a second surgical procedure within the same admission
- Cardiac operations, *defined as any of the following CPT codes, including those with cardiopulmonary bypass: 00560, 00561, 00562, 00563, 00566, 00567, 00580, 50399, 50409, 50410, 50416, 50417, 50714*
- Lung and liver transplant surgery, *defined as any of the following CPT codes: 00796 or 00580*
- Baseline mean arterial blood pressure (MAP) less than 65 *(baseline MAP in holding room MAP or first MAP in operating room if the holding room not available)*
- Cases without documented ICD 9/10 codes
- Pregnant and/or nursing mothers
- Known clinically important intra-cardiac shunts, *defined as ICD 151.0*
- Current persistent atrial fibrillation occurring within the year prior to surgery, defined as any of the following ICD9/10 codes: I48.1, I48.2, or I48.91
- Known acute congestive heart failure, *defined as ICD 9/10 code I50*
- Patients with an intra-aortic balloon pump (IABP) or ventricular assist device(s), *defined as ICD Z95.811*
- Craniotomy, *defined as the presence of any of the following CPT codes: 61304, 61305, 61312, 61313, 61314, 61315, 61320, 61321, 61322, 61323, 61343, 61345, 61458, 61460, 61510, 61512. 61514, 61516, 61518, 61519, 61520, 61521, 61522, 61524. 61526, 61530, 61533, 61534, 61535, 61536, 61537, 61538, 61539, 61540, 61541, 61543, 61544, 61545, 61546, 61548, 61566, 61567, 61570, 61571, 61575, 61576*
- Burn surgery, *defined as the presence of any of the following CPT codes: 16000, 16020, 16025, 16030, 16035, 16036*
- Patient transfer from ICU requiring multiple vasoactive agents and known diagnosis of ongoing active sepsis, *with ICU transferred defined using CPT 99291 and sepsis defined as any of the following codes: A41, A 41.9, A41.02, A41.51, R65.21, postprocedural sepsis (T81.4-); sepsis during labor (O75.3); sepsis following abortion, ectopic or molar pregnancy (O03-O07, O08.0); sepsis following immunization (T88.0); sepsis following infusion, transfusion or therapeutic injection (T80.2-); bacteremia NOS (R78.81); neonatal (P36.-); puerperal sepsis (O85); streptococcal sepsis (A40.-); sepsis (due to) (in) actinomycotic (A42.7); sepsis (due to) (in) anthrax (A22.7); sepsis (due to) (in) candidal (B37.7); sepsis (due to) (in) Erysipelothrix (A26.7); sepsis (due to) (in) extraintestinal yersiniosis (A28.2); sepsis (due to) (in) gonococcal (A54.86); sepsis (due to) (in) herpesviral (B00.7); sepsis (due to) (in) listerial (A32.7); sepsis (due to) (in) melioidosis (A24.1); sepsis (due to) (in) meningococcal (A39.2-A39.4); sepsis (due to) (in) plague (A20.7); sepsis (due to) (in) tularemia (A21.7); toxic shock syndrome (A48.3)*

# Study Exposures and Outcomes

## Exposure

In this amended analysis the primary exposure of interest is whether a case was performed using the HPI technology. In our primary analysis this will be represented by a binary exposure to indicate if the HPI technology was present or absent. If the use of the HPI software was terminated prior to completion of the case, this case will still be analyzed in the HPI group. In the event that a patient was enrolled in the HPI study but did not receive the HPI technology (because of withdrawal or other logistical issue) this case will be marked as not having received the technology.

## Outcomes

The primary outcome is the duration of intraoperative hypotension in minutes. Hypotension will be identified using the MPOG validated blood pressure series that has identified/removed artifacts. The duration of hypotension is defined as the number of minutes in which the mean arterial blood pressure (MAP) is less than 65 for at least one minute.

Presence or absence of acute kidney injury (AKI) will be considered a secondary outcome. AKI will be evaluated using the RIFLE criteria as documented in the electronic health record and uploaded into the MPOG database. Patients are marked as having AKI if they met RIFLE criteria within the first 30 days after surgery. The presence or absence of postoperative nausea or vomiting (as a composite) will be evaluated as a secondary outcome. This will be based on the MPOG phenotype for PONV, which is defined as present if a patient has a documented nausea/emesis occurrence or receives a rescue antiemetic in the immediate postoperative period. Both PONV and AKI will be evaluated as binary variables.

# General Analysis Considerations

## Statistical Principles

All analyses will be performed with pre-specified endpoints and statistical methods. Prior to conducting the analyses, descriptive statistics and data visualizations will be used to characterize the data, the extent of missing values, and to evaluate the distributional assumptions underlying the hypothesis tests.

Statistical assumptions of each model will be considered, including distributions and variance of outcome variables. Means, standard deviations, frequencies, histograms, and levels of skewness and kurtosis will be considered in determining how best to represent variables of interest. If these assumptions are violated, which may be assessed using prescribed tests for such violations, proper adjustments to the model will be considered. Also considered will be the individual nature of each variable of interest as it relates to the specific subject matter.

## Power Analysis

The available data is sufficient to allow meaningful evaluation of the primary hypothesis under study. Assuming that n = 425 participants are able to be analyzed for the HPI group, and that n = 20,000 participants are available for the non-HPI exposure and using two-tailed alpha of 0.05 for an independent t-test, the analysis will have power = 0.80 to detect effect sizes as small as d = 0.14 standard deviation units between the two exposure groups. With an expected mean duration of hypotension < 65 mmHg of 29.3 minutes and an assumed SD = 42, this sample size would allow detection of average differences of 5.9 minutes between exposure groups.

## Descriptive Statistics

Descriptive statistics will be calculated and reported. Continuous data will be reported as mean ± standard deviation or median (interquartile range [IQR]) depending on the distribution of the data. Categorical data will be presented as frequency counts and proportions. Weighted and unweighted standardized mean differences (SMD) will be calculated for patients who did and did not receive the HPI technology. Where appropriate all hypothesis testing will be two-tailed, with p < 0.05 interpreted for statistical significance.

# Primary Analysis

The primary analysis will evaluate the association between the HPI technology and the incidence of intraoperative hypotension, as defined above. To evaluate the difference in hypotension duration conditional on the use of HPI, a generalized linear model will be conducted regressing duration of hypotension on the fixed effects for HPI presence or absence. The model will specify a normal distribution and either an identity or log link to accommodate the distribution under study. Because it is anticipated that patients with longer surgical cases may have an increase period at risk of developing hypotension, the primary model will be adjusted for the duration of intraoperative blood pressure measurement (i.e., time at risk).

It is anticipated that patients may have a different probability of receiving the HPI technology, therefore the primary analysis will use propensity score analyses. Specifically, individuals who elected to participate in the HPI study might possess characteristics that differentiate them from eligible individuals who either declined to participate or were not offered participation. To address this selection bias, a logistic regression model will be developed that predicts HPI participation (i.e., yes vs no) conditional on demographic and disease characteristics. The predicted probability of participation will then be used as an inverse-probability of treatment weight (IPTW) in a second and final model that examines each outcome conditional on HPI use (i.e., yes or no) and duration of blood pressure measurement. Given the inability to differentiate intraoperative confounding in retrospective data because of the temporal relationship with hypotension occurrence and treatment, no other intraoperative covariates will be included in the final model.

# Secondary Analyses

Secondary analyses will be conducted using generalized linear models (GLM). GLM is a flexible analytic tool as it allows for the specification of both categorical and continuous independent and dependent variables. It also allows for the specification of outcome variable distributions (e.g., normal, binomial) and link functions (e.g., identity and log) in case of non-normal data. Using the same structure as the primary analysis, we will model AKI and PONV using a binomial distribution with a log link in separate models.

# Sensitivity and Exploratory Analyses

Several planned sensitivity models will be conducted. First, the influence of missing data on the estimated associations will be examined using multiple imputation. This approach will be conducted to impute exposure, IPTW, or outcome status using the ‘mice’ package in R.

In the event of substantial amounts of missing data (missingness > 10% of available cases), an additional model will be estimated that estimates the association between HPI status and outcome(s) using a multiple regression model (in lieu of IPT weighting) with maximum likelihood estimation.

Additional subgroup analyses may be performed after reviewing observed associations, however these will be denoted as post-hoc in any publication, unless specified above.

# Reporting Considerations

## Reporting Conventions

Whenever necessary study values will be reported in accordance with journal requirements. In lieu of formal requirements for reporting, p-values ≥ 0.01 will be reported to two decimal places, p-values ≥ 0.001 will be reported to three decimal places and p-values less than 0.001 will be reported as “<0.001”. The mean, standard deviation, and any other statistics, will be reported to one decimal place greater than the original data. Estimated parameters, not on the same scale as raw observations (e.g. regression coefficients) will be reported to two significant figures.

## Quality Assurance of Statistical Programming

Given our commitment to conducting reproducible analyses, a copy of all statistical programming code, results and dataset(s) will be maintained by the study biostatistician and the study sponsor. At the start of any code file there will be a set of comments that give (a) the author, (b) the date and time of writing, (c) references to inputs and outputs, and (d) reference to any parent code files, as applicable.

## Statistical Software

All analyses will be conducted using the most recent version of R (http://www.R-project.org/) or RStudio (<http://www.rstudio.com/>).

# Role of the Study Sponsor

This study is sponsored by Edwards Lifesciences, with data analysis completed locally at Massachusetts General Hospital by members of the Anesthesia Research Center (ARC), which is housed within the Department of Anesthesia, Critical Care and Pain Medicine.
