# Supplementary figures and images for: AcumenTM hypotension prediction index guidance for prevention and treatment of hypotension in noncardiac surgery: a prospective, single-arm, multicenter trial
Source: Perioper Med (Lond). 2024 Mar 4;13:13. doi: 10.1186/s13741-024-00369-9 (PMC10913612; doi:10.1186/s13741-024-00369-9)

Supplementary Figure 1. HPI Trial Diagram

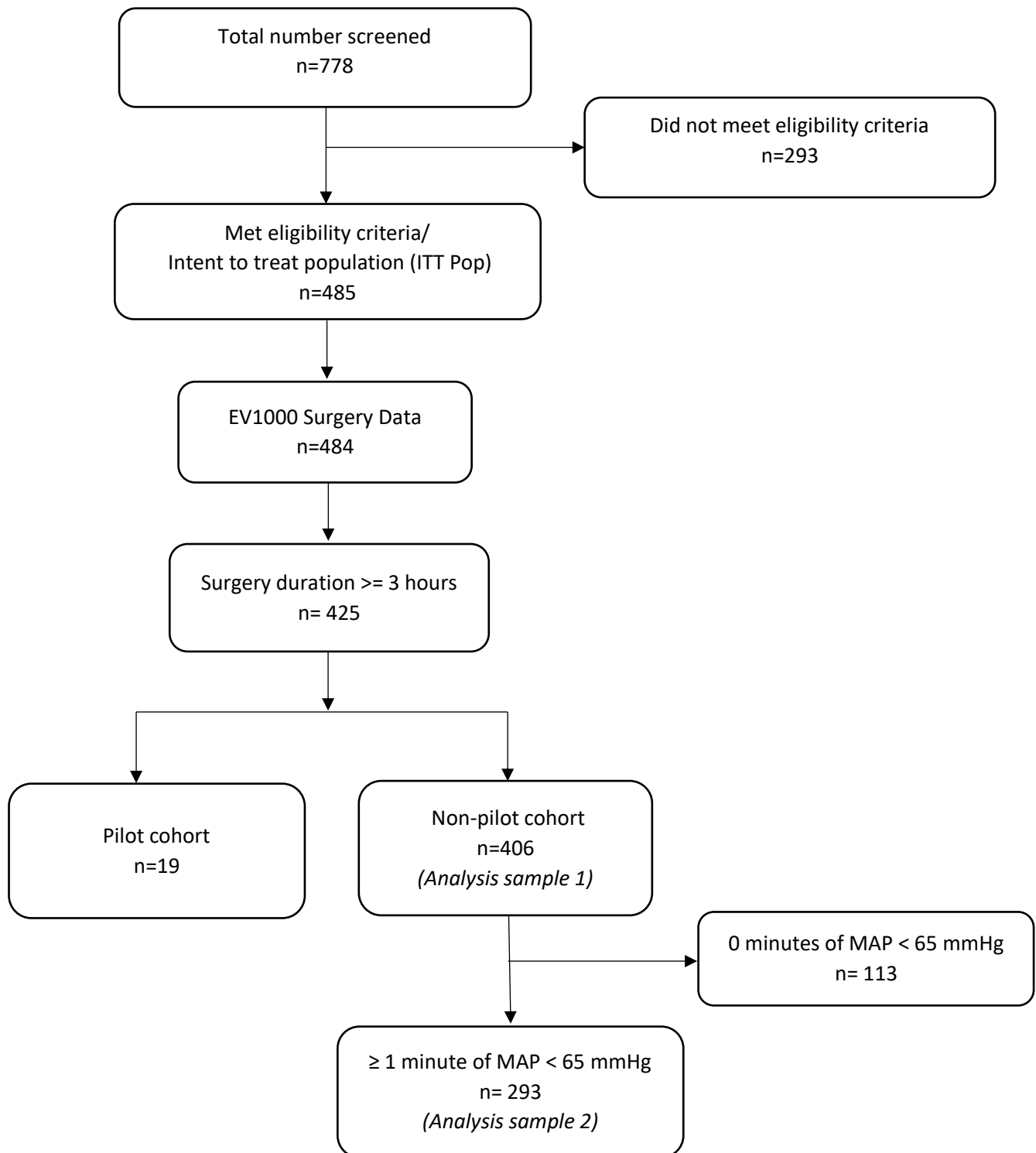

Supplement: Supplementary file 3 — Additional file 3: Supplementary Figure S1. HPI Trial Diagram [file 13741_2024_369_MOESM3_ESM.pdf]

**Figure 2. Data Flow Diagram**

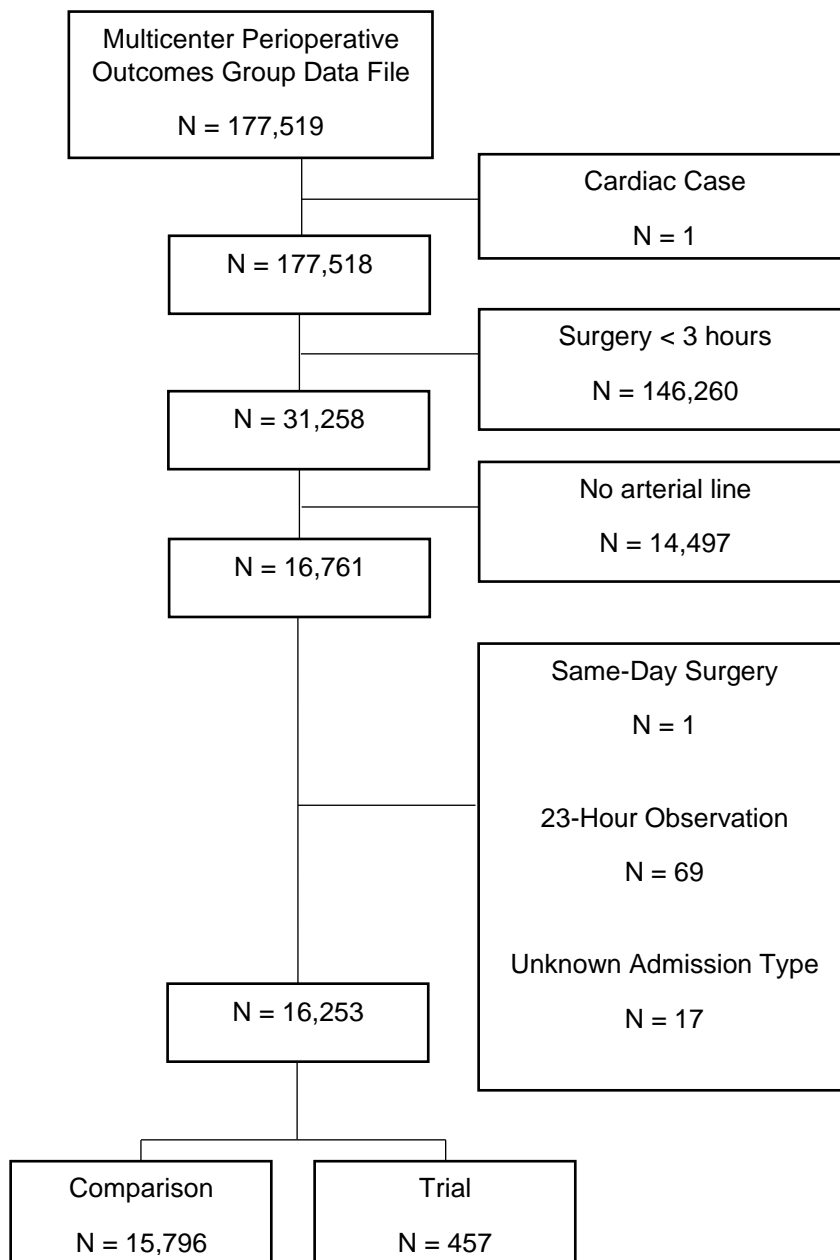

Supplement: Supplementary file 4 — Additional file 4: Supplementary Figure S2. Data Flow Diagram [file 13741_2024_369_MOESM4_ESM.pdf]
